# Family-Centered Care Coordination in an Interdisciplinary Neurodevelopmental Evaluation Clinic: Outcomes From Care Coordinator and Caregiver Reports

**DOI:** 10.3389/fped.2020.538633

**Published:** 2020-10-30

**Authors:** Rebecca McNally Keehn, Brett Enneking, Margo Ramaker, Michael Goings, Ziyi Yang, Aaron Carroll, Mary Ciccarelli

**Affiliations:** ^1^Department of Pediatrics, Indiana University School of Medicine, Indianapolis, IN, United States; ^2^Department of Biostatistics, Indiana University School of Medicine, Indianapolis, IN, United States

**Keywords:** family-centered care, care coordination, neurodevelopmental disabilities, children, interdisciplinary evaluation

## Abstract

Children with neurodevelopmental disabilities experience many unmet healthcare needs. Care coordination is one critical solution to addressing the substantial strain on families, local communities, and the larger healthcare system. The purpose of this study was to implement a care coordination program in an interdisciplinary pediatric neurodevelopmental evaluation clinic and examine care coordinator and caregiver outcomes. Following neurodevelopmental diagnosis, children were provided with either care coordination (CC) or care as usual (CAU). For those receiving CC, the care coordinator documented family goals and care coordination activities, outcomes, and time spent. Caregivers in both groups completed a survey measuring access to needed services and caregiver stress and empowerment following their child's evaluation (T1) and 4–6 months post-evaluation (T2). Care coordinator findings demonstrated that over 85% of family goals focused on understanding the child's diagnosis, getting needed interventions and educational support, and accessing healthcare financing programs. More than half of care coordination activities were spent on engaging and educating the family; similarly, the most time-consuming care coordination efforts were in helping families understand their child's diagnosis and meeting family's basic needs. For those children referred to needed services, 54% were enrolled in one or more service at T2. Caregivers in both the CC and CAU groups reported an increase in stress related to interactions with their child as well as increased empowerment from T1 to T2. Contrary to our hypotheses, there were no significant group-by-time interactions across caregiver-report measures. While these findings further our understanding of care coordination delivery, they diverge from previous evidence demonstrating care coordination efficacy. This study paves the way for future opportunities to evaluate what kinds of care coordination supports family need at varying times in their child's healthcare journey and how the outcomes important to all stakeholders are measured to reflect true evaluation of efficacy.

## Introduction

Children with neurodevelopmental disabilities, including autism spectrum disorder (ASD), developmental delay (DD), and intellectual disability (ID), experience a high level of unmet healthcare needs (1–4). Disparities in access to needed services are greater for children from lower income, minority, and rural families (5–7). Unmet needs are associated with significant family economic (3, 8) and time burden (3), and these challenges are exacerbated for children with greater medical complexity (9). Factors such as increasing access to health insurance that covers needed services, decreasing out-of-pocket spending, reducing exposure to adverse childhood events, improving family employment and financial well-being (2), and creating access to *family-centered and coordinated care* in a medical home (10, 11) have been identified as critical to developing systems of care that address unmet needs and health disparities for children with a variety of special healthcare needs.

Care coordination is one critical solution to addressing the substantial strain that children's unmet healthcare needs place on families, local communities, and the larger healthcare system (12). Care coordination is a “patient- and family-centered, assessment-driven, team-based activity designed to meet the needs of children and youth while enhancing the caregiving capabilities of families. Care coordination addresses interrelated medical, social, developmental, behavioral, educational, and financial needs to achieve optimal health and wellness outcomes” [(13), p. vii]. The American Academy of Pediatrics (14) considers care coordination a standard of care for children with special healthcare needs. Successful care coordination requires a shared focus on health services as well as social determinants of health (12). Further, care coordination programs must cohesively integrate communication and planning among and between children and their families, care coordinators, service providers, community and state agencies, and healthcare payers.

The concept of care coordination is often applied differently across various settings. To address this challenge, McAllister (15) developed a model implementation guide for achieving family-centered, coordinated care for children with special healthcare needs. The foundation of this model rests on the idea that a comprehensive, individualized shared plan of care (SPoC) is the most effective means of driving coordinated, quality, and efficient care. The SPoC is structured into two major components, including a Medical Summary (e.g., description of the child including family composition, diagnosis/problem list, providers in the child's care neighborhood, current and/or past interventions, and pertinent family, social, cultural, or environmental factors or needs) and Negotiated Actions (e.g., including the family and clinical goals with specific actions/strategies, accountable person, and a timeframe for goal completion). Four key elements in the care planning process include: (1) identification of the needs and strengths of the individual and family, (2) building essential partnerships across the care neighborhood, (3) creating, and then (4) implementing the SPoC. Key to successful implementation of the SPoC are communication, collaboration, and co-management among the patient, family, and their care neighborhood. The SPoC must be updated as goals are accomplished, amended, or added and as care of the child evolves over time.

Research has explored the effectiveness of implementing various types of care plans and care coordination programs for children with special healthcare needs, largely in the setting of the medical home. Results of these studies have shown that care coordination has consistently led to improvements in family–professional partnership (16, 17) and family satisfaction (18), as well as reductions in unmet healthcare needs, emergency department visits (19), missed medical appointments (20), inappropriate use of services (21), and family financial and time burden (17, 22).

McAllister and colleagues (23, 24) evaluated the systematic implementation of a 6-month care coordination program in a children's hospital outpatient care setting specifically using the SPoC approach (15) for children with neurodevelopmental disabilities. Results were consistent with significant improvements in care coordination access, SPoC use, family goals achieved, needs met, and family empowerment, as well as reduced caregiver worry (24).

The purpose of the current study was to implement McAllister's (15) SPoC model of care coordination in an outpatient interdisciplinary pediatric neurodevelopmental evaluation clinic and to examine care coordinator and caregiver outcomes. Our first objective was to describe family goals and care coordination activities, outcomes, and time spent as documented by the care coordinator. Our second objective was to compare caregiver-reported changes in unmet service needs, stress, and perceptions of empowerment related to their child's disability between those who received the care coordination (CC) intervention and those who received care as usual (CAU). Based on the findings of McAllister and colleagues (24), we hypothesized that caregivers of children in the CC group would report a greater reduction in unmet needs and stress and increased perceptions of empowerment compared to those receiving CAU.

## Methods

### Participants and Setting

This study was approved by the university Institutional Review Board. Participants were recruited from an outpatient interdisciplinary evaluation clinic at a large Midwestern children's hospital. This clinic is funded by the Maternal and Child Health Bureau's Leadership Education in Neurodevelopmental Disabilities (LEND) program. Children and adolescents, ages 2 through 20 years, are referred to this clinic by their community primary care provider or subspecialist for assessment of neurodevelopmental disabilities (i.e., diagnosis and/or evaluation of neurocognitive profile). The clinic is staffed by an interdisciplinary team of licensed professionals and trainees, including a developmental behavioral pediatrician, clinical psychologist, speech language pathologist, and licensed clinical social worker; an occupational and physical therapist, audiologist, dentist, and dietician are also available.

All children and adolescents receiving neurodevelopmental evaluation in the LEND clinic from fall 2017 through summer 2018 were eligible for participation with the exception of the following exclusionary criteria: (1) children with caregivers who were not fluent in English language, (2) children who were in an unstable caregiving environment at the time of evaluation (i.e., children in custody of the state, recently placed in foster care, or recent history of frequent changes in caregiving environment), and (3) children who were receiving care coordination services through an outside agency at the time of evaluation.

**Figure 1:**
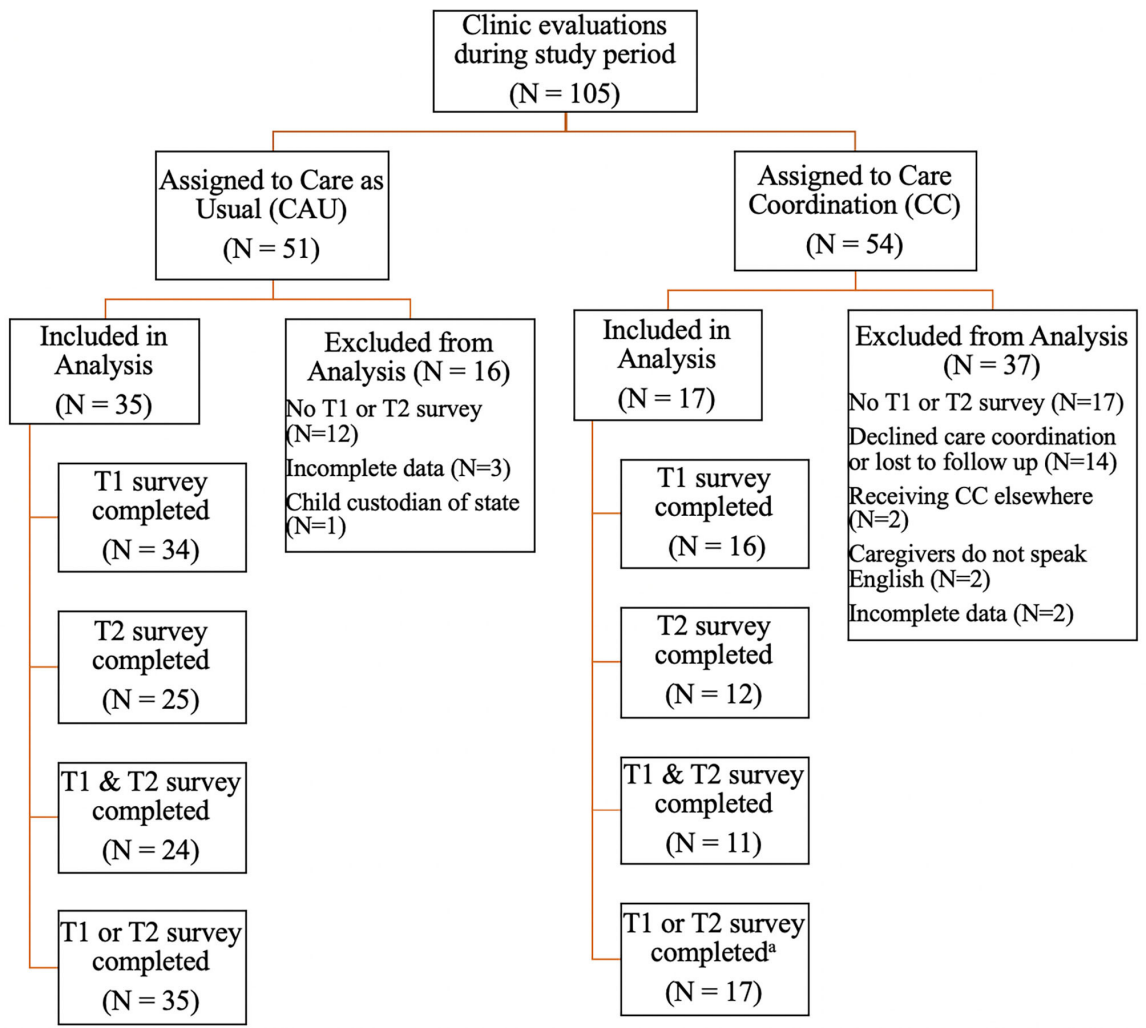
Participant flow through the study. CAU, care as usual; CC, care coordination; T1, Time 1 (baseline); T2, Time 2 (4–6 month follow-up). ^a^The final CC sample (*N* = 17) was used in both the care coordinator report and caregiver report analyses.

A total of 105 children and adolescents were evaluated in this clinic during the study recruitment period. Fifty-four participants were assigned to the care coordination (CC) intervention, and 51 participants were assigned to receive CAU. The final descriptive care coordinator and quantitative caregiver report analysis included 17 participants in the CC group (i.e., those who met inclusion criteria, engaged in the CC intervention, and provided sufficient data for analysis). Thirty-five participants in the CAU group were included in the final quantitative caregiver report analysis (i.e., those who met the inclusion criteria and provided sufficient data for analysis) (see Figure 1 for details of participant flow through the study).

### Intervention Group Assignment and Study Design

Children referred to the interdisciplinary evaluation clinic were assigned to an intervention group (CC or CAU) via a quasi-random process. Specifically, children scheduled into two designated clinic slots per week were provided with the care coordination (CC) intervention, and children scheduled into the remaining two designated clinic spots were provided care as usual (CAU). Selection of clinic slots was based on availability and caregiver scheduling preferences; clinic schedulers and participants' caregivers had no knowledge of the study (including group assignment process) at the time of scheduling. However, the evaluation team (including care coordinator) was not blind to intervention group assignment or to the aims of the research study.

An observational approach was utilized to gather descriptive data on family goals and care coordination activities, outcomes, and time spent on intervention implementation (as documented by the care coordinator) for those receiving CC. A quantitative repeated measures design was employed in order to compare caregiver-reported changes from evaluation (T1) to 4–6 months follow-up (T2) in unmet service needs, stress, and perceptions of empowerment related to the child's disability between those who received CC and those who received CAU.

### Care Coordinator Report Measures

#### Family Goals

Together, the caregiver(s) and care coordinator set care coordination goals during the neurodevelopmental evaluation and throughout the intervention period. Each goal was coded by the care coordinator into one of seven categories: Getting Needed Interventions, Understanding the Diagnosis, Getting Needed Education Plan, Accessing Needed Healthcare, Complex Care Access/Communication, Family Quality of Life, and Meeting Basic Needs [see (23, 24) for a review of the development of goal categories and definitions].

#### Care Coordination Activities

At study initiation, the care coordinator developed a set of care coordination activity categories. Activities completed to address each goal were coded into the following categories: Identification of Needs and Goal Setting (e.g., identifying needs, developing family goals, and developing an action plan), Family Engagement (e.g., building rapport and trust, promotion of therapeutic processing of child's diagnosis, and use of motivational interviewing and solution-focused strategies to engage family in intervention), Education of the Care Neighborhood (i.e., education of the primary care team around procedures for meeting common neurodevelopmental needs, family social determinants of health, and barriers to accessing medical care), Education of the Family (e.g., education on appropriate interventions and local resources pertinent to child's diagnosis and evaluation results), Team Communication (e.g., sharing progress and problem solving, and documentation of activities and progress), and Research and Information Gathering (e.g., gathering information about standards of care for medical diagnoses and resources and referrals for family and care neighborhood). The documentation system allowed for selection of multiple activities in support of each goal.

#### Care Coordination Outcomes

At study initiation, the care coordinator developed a set of outcome categories. For each activity, outcomes across the following categories were recorded: Education Provided (e.g., provided formal psychoeducation and/or training to family, primary care team, or others in care neighborhood), Safety Resources Provided (e.g., facilitated visit to the hospital-based Safety Store and provided specialized safety kit), Enrollment in Healthcare Financing Program (e.g., disseminated required documentation to healthcare financing agency and assisted family in completing healthcare program applications or changing plans to access needed services), Referred for Needed Service (e.g., made referral to medical specialist, therapeutic/intervention agency, or community organization), and Enrolled in Needed Service (e.g., received notice that child was enrolled in needed service). The documentation system allowed for the selection of multiple outcomes (within and across categories) for each goal.

#### Time Spent on Care Coordination

The care coordinator recorded the amount of time (in minutes) spent on care coordination activities completed in support of each family goal. Time spent included direct contact with the caregiver and the child's care neighborhood, as well as time gathering information and resources and completing documentation.

### Caregiver Report Measures

#### National Survey on Children With Special Healthcare Needs [NS-CSHCN 2009–2010; (25)]

The NS-CSHCN 2009–2010 includes items under the category of “Access to Care: Utilization and Unmet Needs.” Seventeen items relevant for children with neurodevelopmental disabilities were adapted to assess unmet needs. Each item is composed of two parts: “During the past 12 months, was there any time when your child needed [physical, occupational, or speech therapy]?” and “Did child receive all the [physical, occupational, or speech therapy] he/she needed?” No known published psychometric data on this NS-CSHCN item set is available. Variables calculated included: total services needed, total services received, and percent unmet needs (sum unmet needs/sum needs).

#### Parenting Stress Index, Fourth Edition, Short Form [PSI-4-SF; (26)]

The PSI-4-SF is a 36-item measure of parenting stress. Items are scored on a five-point scale and grouped into three subscales: Parental Distress, Parent–Child Dysfunctional Interaction, and Difficult Child. A Total Stress score is also derived. PSI-4-SF T-scores were used for the current analysis. The PSI-4-SF has demonstrated strong psychometric properties (27, 28) and has been widely used with demographically (29, 30) and diagnostically diverse populations (31, 32).

#### Family Empowerment Scale [FES (33)]

The FES is a 34-item rating scale developed to measure empowerment in families of children with disabilities. Items are scored on a five-point scale. Two subscales of the FES were included in this study: About Your Family (item examples: “I know what to do when problems arise with my child; I have a good understanding of my child's disorder”) and About your Child's Services (item examples: “I know the steps to take when I am concerned my child is receiving poor services; I am able to work with agencies and professionals to decide what services my child needs”). The FES has demonstrated sound psychometric properties (33) and has been successfully employed in intervention and service outcome research (34, 35).

### Intervention

#### Care Coordination Intervention

McAllister's (15) Shared Plan of Care (SPoC) approach to care coordination was followed. Guiding principles and activities of the care coordination intervention can be found in McAllister (15) and McAllister et al. (23). An overview of the care coordination intervention and procedural differences to this approach are described below. All care coordination activities were overseen and delivered by the clinic's care coordinator, a Licensed Clinical Social Worker, with several decades of experience working with children with neurodevelopmental disabilities and their families and over 3 years of training and experience in implementing McAllister's (15) SPoC model prior to this study.

Prior to a child's neurodevelopmental evaluation, the SPoC was prepopulated by an evaluation team member (e.g., psychology or speech language pathology fellow) with information obtained from medical and collateral records. This information was presented to the interdisciplinary team during a weekly meeting where the child's case was reviewed, and preplanning for potential care coordination needs occurred with cross-discipline input. The SPoC included (1) a Medical Summary detailing information about the child and family (i.e., contact information, who resides in the home, language(s) spoken, family stressors, and preferences), significant developmental history, insurance and related healthcare financing programs, educational programs and supports, primary and specialty care providers, medical diagnoses and conditions, hospitalizations, surgeries and procedures, and family medical history, and (2) Family Goals with information detailing the action steps and overall plan for addressing identified unmet needs.

During the evaluation, the care coordinator conducted a biopsychosocial interview with the child's caregiver(s) to establish rapport and trust, orient them to the process of care coordination, learn about the child and family, and identify unmet needs and develop initial care coordination goals. Goals were derived from needs identified by caregivers via a collaborative discussion between the child's caregivers and the care coordinator. Reflective listening, motivational interviewing, and solution-focused strategies were employed by the care coordinator during the interview, as well as during the follow-up period of active care coordination. Families received the SPoC with initial family goals as well as clinical recommendations and printed resources pertinent to the results of the neurodevelopmental evaluation at the conclusion of the evaluation.

Caregiver(s) received an initial phone contact by the care coordinator within 2 weeks following their child's evaluation during which family goals were reviewed, amended, and prioritized, and action steps were set. The coordinator contacted the child's primary care provider's office to review the SPoC and results of the neurodevelopmental evaluation as well as communicate any requests for the PCP. Often this communication occurred between the care coordinator and a medical assistant or nurse at the PCP's office. While the primary care team was invited to provide feedback about the SPoC and make requests of the coordinator, often this contact resulted in the care coordinator educating the primary care team about how to meet the common needs of children with neurodevelopmental disabilities (e.g., completing referrals for adaptive car seat, prescription for incontinence supplies, etc.). When indicated, resources and educational materials specific to the needs of the individual child or general to individuals with neurodevelopmental disabilities were provided to the primary care team.

The frequency and intensity of care coordination varied depending on family engagement and capacity, urgency of the unmet need(s), responsivity of the care neighborhood, and availability of needed services. Most contact with families and the care neighborhood was completed by phone, email, and fax, though some families did return to the hospital for other medical needs, and in-person meetings with the coordinator were conducted. The coordinator also worked with members of the child's neurodevelopmental evaluation team to gather clinical recommendations and information, as well as problem solve around barriers to meeting needs. The SPoC was updated by the care coordinator and disseminated to each family and their child's relevant care providers as goals were achieved, amended, and/or added.

#### Care as Usual

The care coordinator conducted a biopsychosocial interview with the child's caregivers during the course of the child's neurodevelopmental evaluation. Families were provided with the Medical Summary portion of the SPoC as well as clinical recommendations and printed information and resources relevant to the child's diagnosis at the conclusion of the evaluation. Caregivers were contacted by phone by a member of the study team within 3 weeks following their child's evaluation to answer any questions about the diagnosis or recommendations made during the evaluation. No further support was offered.

### Procedure

On the day of each child's neurodevelopmental evaluation, caregivers were asked if they were interested in participating in a study about children's neurodevelopmental needs and services. If interested, they were provided with verbal information about the study as well as an IRB-approved Study Information Sheet detailing the study purpose, procedures (including those in place to protect confidentiality), risks and benefits to participation, and participant reimbursement procedures. All participating caregivers, regardless of intervention group assignment, received a caregiver report survey (i.e., Caregiver Report Measures) on the evaluation day (T1) as well as 4–6 months following their child's initial neurodevelopmental evaluation (T2). Surveys were completed via paper/pencil (and returned via paid envelope) or link to an individual, confidential online data collection tool (i.e., REDCap). Participants received a $20 gift card for each completed survey.

The care coordinator systematically documented information about provision of the care coordination intervention for each participant receiving this service (i.e., family goals, intervention activities, intervention outcomes, and time spent on the intervention) in a secure online research database throughout the data collection period (i.e., over the course of 4–6 months). No information was documented by the care coordinator for those in the CAU group.

### Statistical Analysis

For observational care coordinator report outcomes, descriptive statistics (i.e., frequencies and means) were calculated. For the quantitative analysis, group differences in demographic variables were analyzed using a two-sample *t*-test for continuous variables and Fisher's exact or chi-square test for categorical variables. A linear mixed model was employed to examine main effects and group by time interactions [i.e., group (CC; CAU) + time (T1; T2) + group × time with the random intercepts] for caregiver report measures. This model not only incorporated the correlation of repeated measures for the same participant but also accounted for missing values with the assumption of missing at random. Statistical analyses were performed in SAS version 9.4.

## Results

### Care Coordinator Report Outcomes

**Figure 2:**
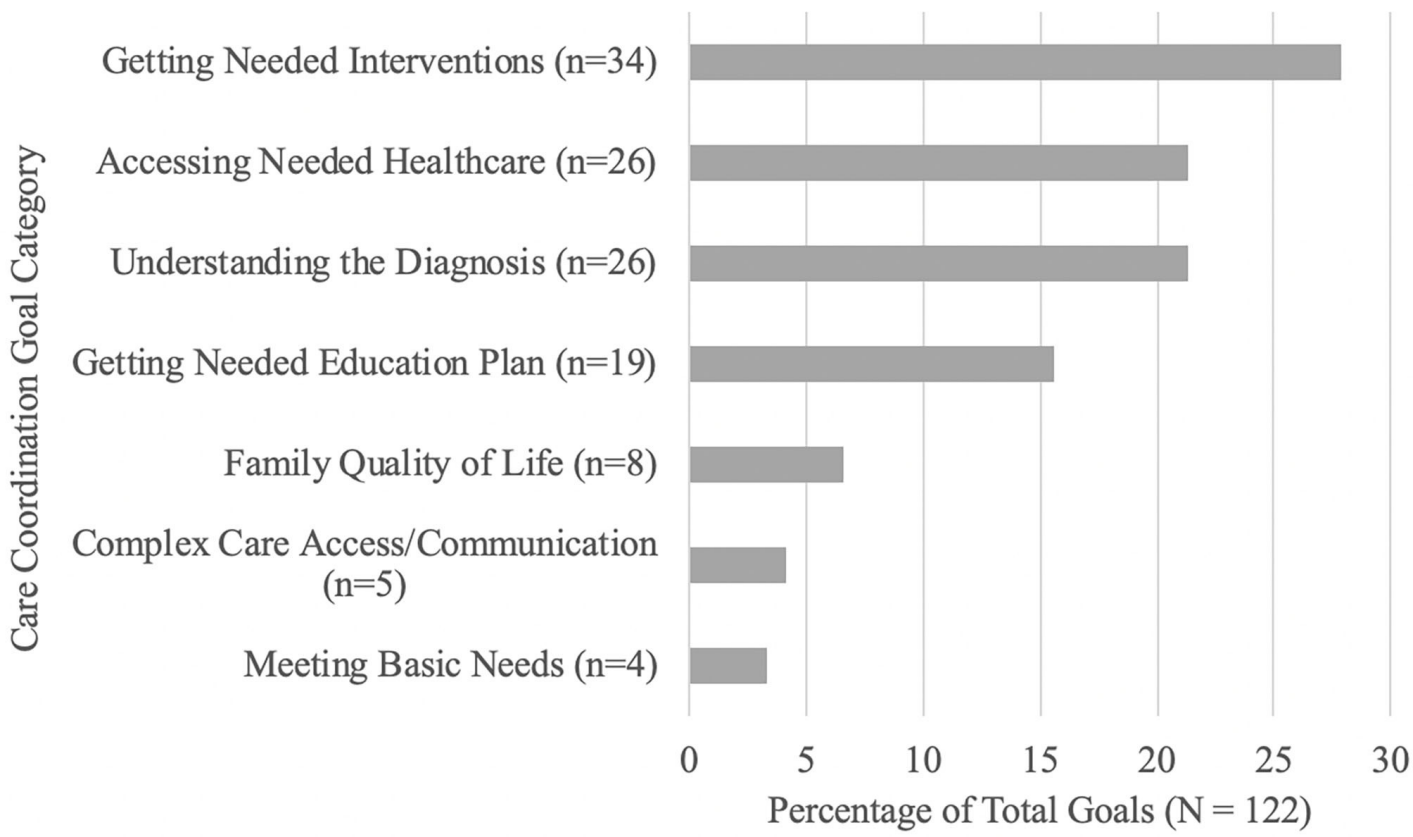
Care coordination family goals.

Together, families and the care coordinator generated a mean of 7.06 (SD = 4.62) goals during the care coordination period. Getting Needed Interventions (27.87%), Accessing Needed Healthcare (21.31%), Understanding the Diagnosis (21.31%), and Getting a Needed Education Plan (15.57%) accounted for over 85% of family goals. Improving Family Quality of Life accounted for 6.56% of goals, while Complex Care Access/Communication accounted for 4.10% and Meeting Basic Needs accounted for 3.28% of goals (see Figure 2).

**Figure 3:**
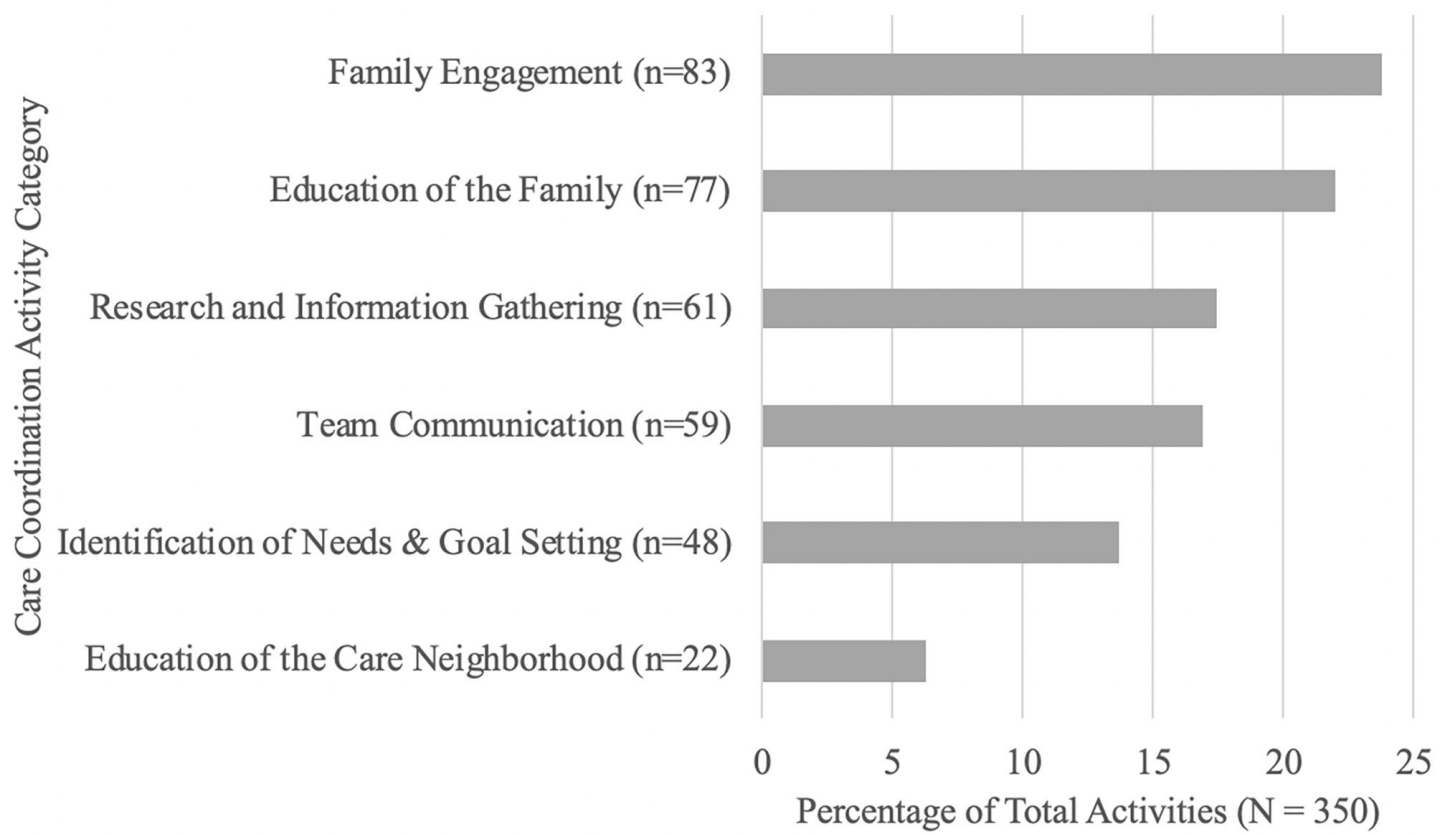
Care coordination activities.

Together, Engagement (23.71%) and Education (22.00%) of the Family accounted for half of care coordination activities. Nearly 18% of activities focused on Research and Information Gathering (17.43%), while 16.86% of activities focused on Team Communication (i.e., within the clinic team), 13.71% of activities focused on Identification of Needs and Goal Setting, and 6.29% of activities focused on Education of the Care Neighborhood (see Figure 3).

**Figure 4:**
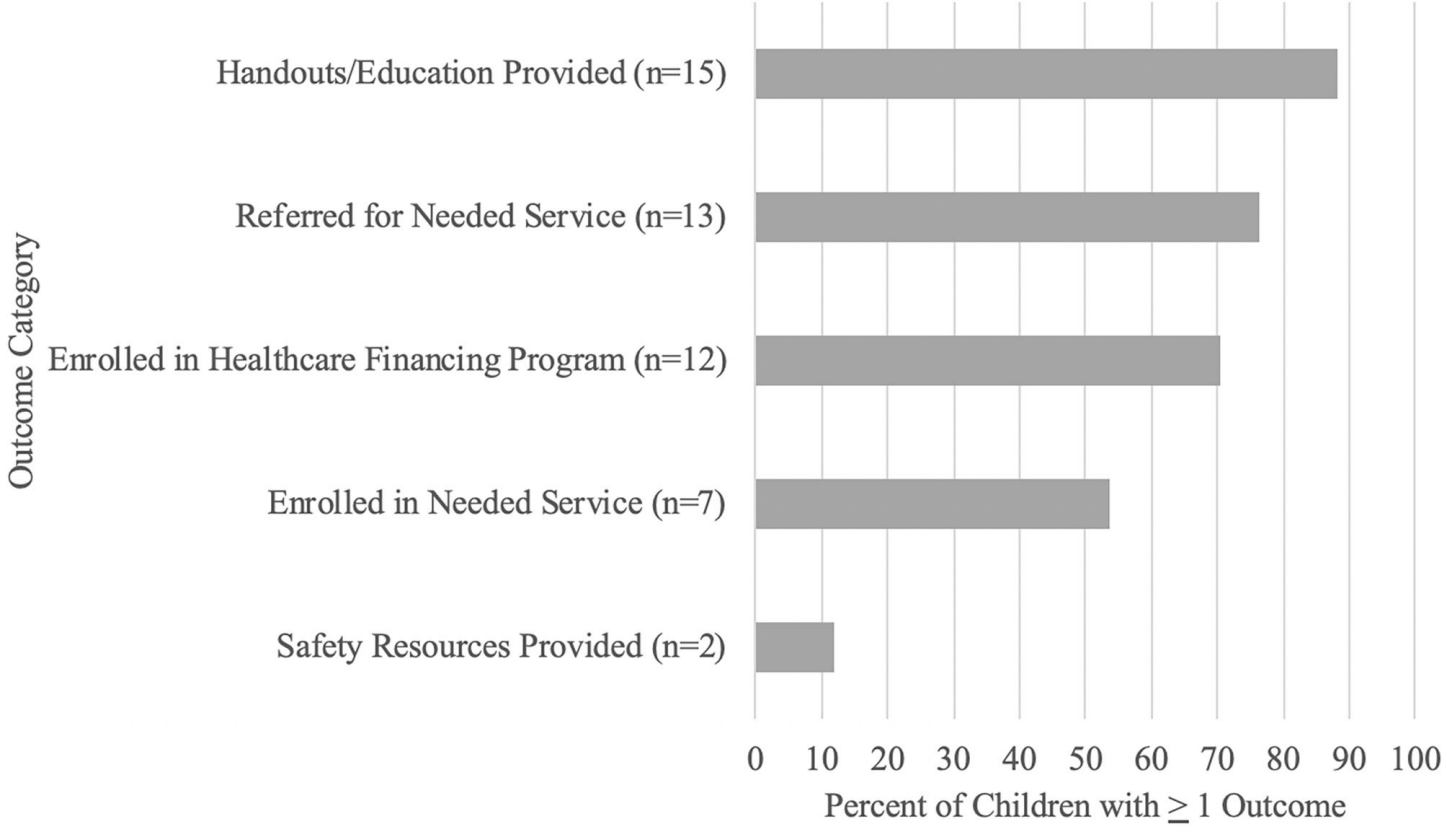
Care coordination outcomes.

A significant majority of children's families and/or care neighborhood (i.e., 88.20%) received formal psychoeducation or educational materials to support achievement of care coordination goals. Similarly, 76.50% of children were referred to one or more needed services and, of those children referred to a service (*n* = 13), 53.80% (*n* = 7) of the children were enrolled in a needed service by T2. Seventy percent (i.e., 70.60%) of children were enrolled in one or more healthcare financing programs, and 11.8% of the children received safety resources (see Figure 4).

**Figure 5:**
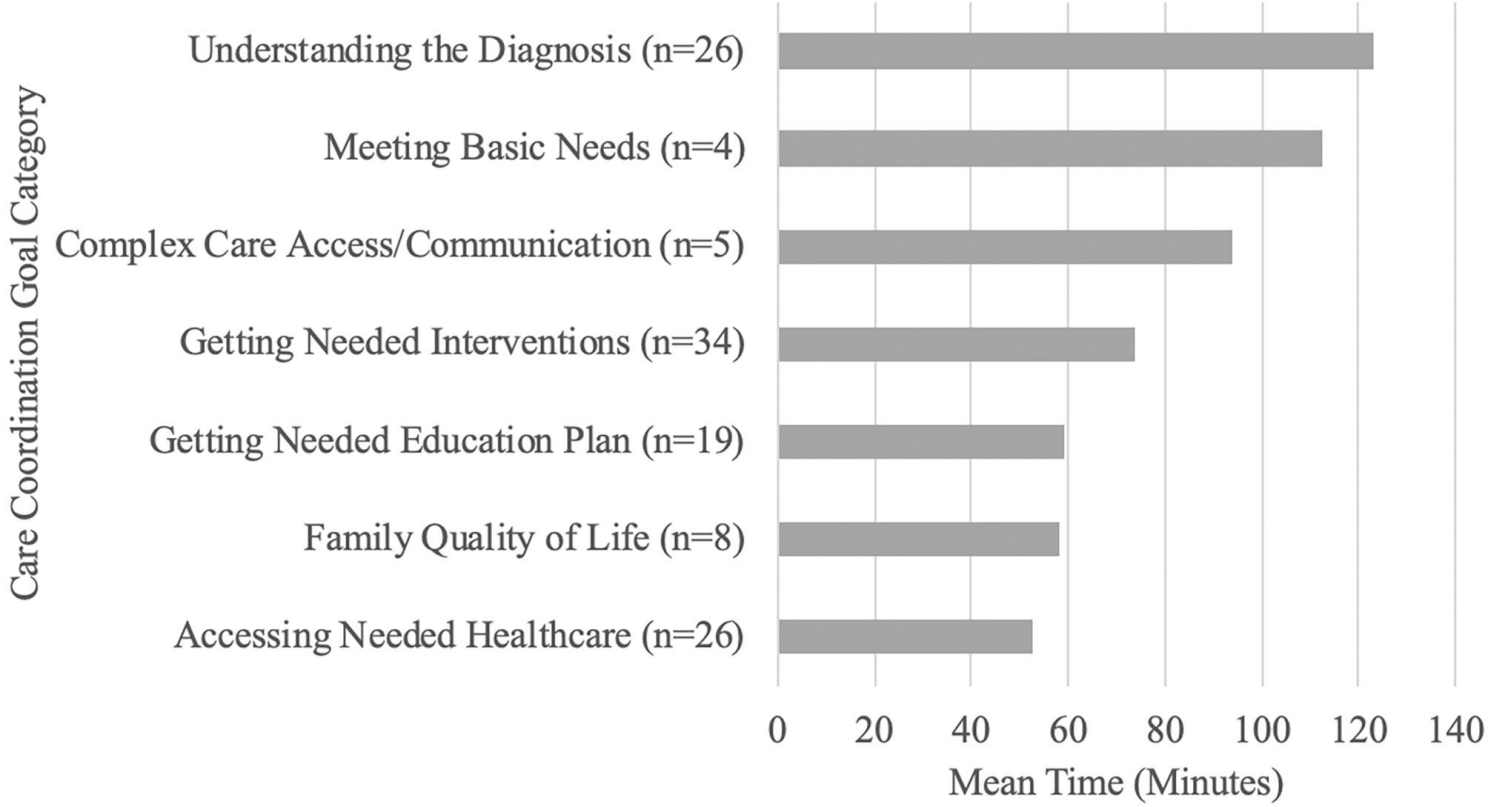
Time spent on care coordination.

A mean of 9.35 h (561.24 min; SD = 312.76) was spent in care coordination per child for the 4- to 6-month duration of the intervention. The most time-consuming care coordination activities were focused on goals related to Understanding the Diagnosis (mean: 123.23; SD: 79.50), Meeting Basic Needs (mean: 112.50; SD: 175.05), Complex Care Access/Communication (mean: 94.00; SD: 35.60), and Getting Needed Interventions (mean: 73.77; SD: 55.84). The care coordinator spent nearly 1 h in activities to support each of the other goal categories: Getting Needed Education Plan (mean: 59.26; SD: 36.08); Family Quality of Life (mean: 58.13; SD: 30.70); and Accessing Needed Healthcare (mean: 52.42; SD: 29.02) (see Figure 5).

### Caregiver Report Outcomes

Table 1 summarizes the demographic characteristics for participants who completed a caregiver report survey at one or more time points. There were no significant differences in demographic variables between CC and CAU groups. Table 2 displays summary statistics (mean and SD) for caregiver report outcomes by group and time; *p*-values are provided for comparisons of difference in change from T1 to T2 between CC and CAU groups.

**Table 1:** Participant demographic characteristics by intervention group.

	**CAU (*N* = 35)**	**CC (*N* = 17)**	***p***
Age in years [mean (SD)]	6.0 (2.4)	5.7 (2.8)	0.68
Sex (*N*; %)			>0.99
Male	10 (28.6)	4 (23.5)	
Female	25 (71.4)	13 (76.5)	
Race (*N*; %)			0.57
White	27 (79.4)	14 (82.4)	
Black or African American	3 (8.8)	3 (17.6)	
More than one race	3 (8.8)	0 (0)	
Unknown	1 (2.9)	0 (0)	
Ethnicity (*N*; %)			>0.99
Non-Hispanic or Latino/a	32 (94.1)	16 (100.0)	
Hispanic or Latino/a	2 (5.9)	0 (0)	
Diagnosis (*N*; %)			0.96
Autism spectrum disorder	12 (35.3)	6 (35.3)	
Developmental delay/intellectual disability	5 (14.7)	3 (17.6)	
Other neurodevelopmental disability	17 (50.0)	8 (47.1)	

*All NIH designated race categories were included as options; only those represented in the participant sample are included above. Other neurodevelopmental disability included diagnoses such as language disorder and attention deficit hyperactivity disorder. CAU, care as usual; CC, care coordination*.

**Table 2:** Caregiver report measures: Group by time summary statistics.

**Outcome measure**		**CAU mean (SD)**		**CC mean (SD)**		***p*^**a**^**
		**Time 1**	**Time 2**	**Time 1**	**Time 2**	
NS-CSHCN	Total services needed	5.22 (1.95)	4.93 (2.34)	4.81 (2.81)	6.43 (3.48)	0.14
	Total service received	4.16 (1.59)	4.14 (1.88)	3.69 (2.27)	4.29 (2.37)	0.54
	Percent unmet needs	31.48 (13.64)	32.02 (11.54)	35.80 (11.48)	43.11 (22.09)	0.28
PSI-4-SF	Parental distress	69.74 (10.78)	70.18 (9.96)	69.81 (8.38)	72.50 (9.04)	0.35
	Parent–child dysfunctional interaction^b^	72.68 (7.40)	73.86 (7.27)	69.88 (10.49)	74.50 (8.22)	0.17
	Difficult child	61.82 (8.64)	62.89 (8.80)	61.25 (9.78)	62.86 (10.35)	0.44
	Total stress	69.64 (8.24)	70.75 (8.19)	68.75 (9.38)	71.93 (9.14)	0.24
FES	About your family	47.91 (7.50)	50.00 (6.13)	50.19 (6.52)	50.21 (6.57)	0.45
	About your child's services^b^	49.44 (10.17)	53.00 (6.39)	50.00 (7.90)	53.36 (6.55)	0.95

*NS-CSHCN, National Survey on Children with Special Healthcare Needs 2000–2010 (Access to Care: Utilization and Unmet Needs); PSI-4-SF, Parenting Stress Inventory, Fourth Edition (T-scores), Short Form; FES, Family Empowerment Scale; CAU, care as usual; CC, care coordination*.

a *p-value represents the significance of difference from T1 to T2 between two groups (generated via linear mixed model)*.

b *Significant main effect of time (p < 0.05)*.

There were no significant main effects for group or time for any of the three unmet needs outcome variables, including the total number of services needed, total number of services received, and percentage of unmet needs (all *p* > 0.05). Similarly, there were no significant group by time interactions for total number of services needed, *F*_(1,31)_ = 2.26, *p* = 0.14, total number of services received, *F*_(1,31)_ = 0.39, *p* = 0.53, or percentage unmet needs, *F*_(1,17.7)_ = 1.26, *p* = 0.28, as measured by the adapted NS-CSHCN Unmet Needs items. However, caregivers who received the CC intervention (T1 mean: 4.81, SD: 2.81, T2 mean: 6.43, SD: 3.48) reported an increase in services needed from T1 to T2 relative to those in CAU (T1 mean: 5.22, SD: 1.95, T2 mean: 4.93, SD: 2.34).

There was a significant main effect of time for PSI-4-SF Parent–Child Dysfunctional Interaction T-scores, *F*_(1,42.7)_ = 4.96, *p* = 0.03, such that caregivers in both groups evidenced higher stress related to challenging interactions with their child at T2. No additional main effects of time or group were found for any other PSI-4-SF outcomes (all *p* > 0.05). There were no significant group by time interactions for any of the PSI-4-SF outcomes, including Parental Distress T-score, *F*_(1,38.1)_ = 0.91, *p* = 0.35, Parent–Child Dysfunctional Interaction T-score, *F*_(1,42.7)_ = 1.94, *p* = 0.17, Difficult Child subscales T-score, *F*_(1,40.6)_ = 0.61, *p* = 0.44, and Total Stress T-score, *F*_(1,40)_ = 1.41, *p* = 0.24.

There was a significant main effect of time for FES About Your Child's Services scores, *F*_(1,48)_ = 4.71, *p* = 0.04, indicating that all caregivers perceived an increase in knowledge and agency about the services their child received from T1 to T2. There were no additional significant main effects for time or group for other FES outcomes (all *p* > 0.05). There were no significant group by time interactions for FES outcomes, including About Your Family, *F*_(1,35.6)_ = 0.59, *p* = 0.45, and About Your Child's Services, *F*_(1,48)_ = 0.00, *p* = 0.95.

## Discussion

Our objective was to integrate a model care coordination program into an interdisciplinary pediatric neurodevelopmental evaluation clinic and to (1) describe family goals and care coordinator reported activities, outcomes, and time spent implementing the care coordination intervention and (2) examine whether those provided with care coordination following McAllister's (15) Shared Plan of Care model demonstrated greater benefit with regard to access to needed services and other family reported outcomes than those provided with care as usual.

Results of care coordinator report outcomes revealed that over 85% of family care coordination goals following neurodevelopmental evaluation focused on understanding the child's diagnosis, getting needed interventions and educational support, and accessing healthcare financing programs to support these needs. Despite the differential timing of when care coordination was initiated (i.e., months or years following neurodevelopmental evaluation/diagnosis vs. immediately following evaluation/diagnosis), our findings align with those of McAllister and colleagues (24) in that families of children with neurodevelopmental disabilities prioritize accessing and financing needed interventions.

To our knowledge, this is the first study to measure care coordination activities and time spent for children with neurodevelopmental disabilities. Care coordination is a time-consuming intervention. While there was significant variability in time spent across families, likely related to family psychosocial complexity (36), on average, over 9 h was spent addressing family goals during the 4- to 6-month intervention period. Our care coordinator spent the greatest amount of time helping families understand their child's diagnosis and meeting basic family needs. The most frequent activities to support these goals were engaging and educating the family. Further, nearly 90% of the families and/or the child's care neighborhood received formal psychoeducation and educational materials to support these efforts. These findings speak of the critical importance of supporting families in understanding, processing, and actively engaging in efforts around their child's diagnosis immediately following neurodevelopmental evaluation. Overall, our findings are largely consistent with previous research highlighting that much of care coordination is focused on navigating non-medical needs (24, 36–38).

Based on care coordinator report, over 75% of children were referred to one or more needed service, and of those referred, over 50% were enrolled in one or more services by T2. However, contrary to expectations, previous reports [e.g., (18, 24, 39)], and our own care coordinator report outcomes, we did not find that caregiver reported unmet needs decreased following engagement in care coordination. We found that those receiving care coordination reported an increase in number of services needed over time, suggesting that caregivers learned what services their child needed but were not yet receiving at T2. It is possible that our discrepant care coordinator and caregiver report findings can be attributed to differences in the two measurement systems. While our care coordinator documented enrollment in any needed service, the caregiver report NS-CSHCN Unmet Needs items focus on specific domains and ask whether the child is receiving *all* the needed services in that domain. As such, our care coordinator report data may have captured a broader range of services. Further, although a similarly adapted NS-CYSHCN item-set [e.g., (24)] demonstrated sensitivity to change over time, differences in our methodology (i.e., shorter follow-up period) may have precluded the ability to detect meaningful change as a result of the care coordination intervention.

Our results regarding unmet needs and service enrollment should be understood in the context of the geographic setting and the complex sociocultural environment of the Midwest. For example, this study was conducted in a region in which services are often sparse, and families are faced with long waitlists (i.e., often 6–12-months or more). Although our neurodevelopmental clinic has a long history of building cross-sector partnerships that support care access for the families that we serve, the systems are overburdened and underresourced, and it is likely that local and regional services were often not available and/or waitlists for enrollment exceeded the 4- to 6-month follow-up period. It must be also considered that, even when provided referrals for needed services, not all families may be emotionally ready to seek recommended interventions immediately following their child's diagnosis. Moreover, similar to prior studies (36, 40), families in our study were faced with challenging social determinants of health barriers (i.e., accessing adequate food, housing, and transportation; family mental health and legal support needs). Our evaluation clinic provides services to large numbers of children receiving Medicaid insurance, residing in rural communities, and involved in child protective services and these factors cannot be overlooked when interpreting our findings. Substantial care coordination efforts and time were focused on addressing these unmeasured barriers prior to shifting focus and resources to the child's service needs.

While previous studies have shown that care coordination is associated with reduced caregiver worry (24), we did not find that care coordination ameliorated global or specific measures of caregiver stress. Instead, our results suggested that caregivers across both groups reported an increase in stress related to interactions with their child in the 4- to 6-months following neurodevelopmental diagnosis. These findings are supported by an extensive literature regarding high levels of stress in caregivers of children with ASD and other neurodevelopmental disabilities [see (41) for review].

Care coordination aims to build caregiver capacity (13) to address individual- and family-level needs and to seek collaboration and assistance from the care neighborhood when these needs cannot be met independently. Single-group studies of care coordination (24) have documented increased family empowerment. Our findings suggest that caregivers across both groups reported high levels of baseline empowerment, and empowerment regarding understanding and advocacy for services increased over time. It may be that the process of engaging with a family-centered, strength-based interdisciplinary team during the child's neurodevelopmental evaluation facilitated knowledge and confidence regardless of receipt of care coordination.

Several important limitations to the study warrant discussion. First, while the care coordinator had a high level of experience in delivering McAllister's (15) SPoC model of care coordination, we did not measure fidelity to the intervention model. Second, intervention group assignment was not randomized. While no group differences were found in any demographic variables for those who completed at least one caregiver report survey, the study design cannot rule out differences in demographic variables between those who did and did not complete the survey. We found a 33% return rate on caregiver surveys and, as such, results may not be representative of all families who were evaluated in our clinic or received care coordination services. It should be noted that a higher proportion of participants in the care coordination group, compared with those receiving care as usual, did not return a caregiver-report survey at either time point or were excluded for other reasons (i.e., declining or not following through with the intervention). Third, our sample size was small, and thus, statistical power may have been limited, and results must be interpreted with caution. Fourth, and of most potential significance, is the limitation in interpretation of findings due to the short duration of the data collection period. Given the shortage of service availability and resulting long waitlists, 4–6-months is likely not long enough to assess whether children and family service needs are being met as a result of the intervention. Finally, the quantitative outcome measures utilized did not capture the fundamental social determinants of health-related goals, activities, and outcomes that were documented by the care coordinator. More comprehensive measurement of demographic factors that may influence outcomes, as well as utilization of outcome measures sensitive to social determinants of health may further contextualize findings from future care coordination studies. In addition, measurement of family and provider satisfaction may have provided a complementary, and perhaps more robust, measure of intervention success than the measurement tools used in the current study.

In conclusion, an emerging body of research has demonstrated the effectiveness of care coordination for a broad array of important family and health outcomes, including improvement in family–professional partnership and satisfaction, reduction in family time and financial burdens, reduction in unmet needs, and inappropriate use of the healthcare system. Despite these positive benefits, care coordination is a complex, time- and skill-intensive process for which measuring outcomes proves challenging. Our findings suggest that families of children with neurodevelopmental disabilities prioritize accessing and financing needed interventions. Yet, the activities of care coordination, at least immediately following initial neurodevelopmental evaluation, must largely focus on providing support and education to the child's family and care neighborhood before more directly measurable benefits may be realized. Four to 6 months is not long enough for most children with neurodevelopmental disabilities to access needed services in a geographic area with substantial service shortages. Further, it may not be enough time to support the reduction of caregiver stress and meaningfully bolster empowerment and advocacy skills. While care coordinator report findings further our understanding of the delivery of care coordination for children with neurodevelopmental disabilities, our caregiver report findings diverge from the emerging body of strong evidence for the efficacy of care coordination. However, we believe they pave the way for future opportunities to further evaluate what kinds of care coordination supports families need at varying times in their child's healthcare journey and how the outcomes important to all stakeholders are measured to reflect true evaluation of efficacy. Future studies should focus on the use of a randomized controlled trial design and development of sensitive tools to measure the outcomes important to all involved in the process: families, providers, and the systems that they operate within and across.

## Data Availability Statement

The data analyzed in this study are not publicly available due to Human Subjects protections. Requests to access the datasets should be directed to Rebecca McNally Keehn (mcnallyr@iu.edu).

## Ethics Statement

The studies involving human participants were reviewed and approved by The Indiana University IRB. Patient's/participant's written informed consent to participate was not required for this study in accordance with institutional guidelines.

## Author Contributions

RM and BE conceptualized and designed the study, contributed to designing the data collection instruments, data collection, analysis, and interpretation. MR contributed to designing the data collection instruments and data collection and interpretation. ZY and MG contributed to data analysis and interpretation. MC and AC provided critical intellectual feedback at all stages of the study. All authors reviewed and revised the manuscript for important intellectual content, approved the final manuscript, and agreed to be accountable for all aspects of the work.

## Conflict of Interest

The authors declare that the research was conducted in the absence of any commercial or financial relationships that could be construed as a potential conflict of interest.
